# Human endogenous retroviruses and exogenous viral infections

**DOI:** 10.3389/fcimb.2024.1439292

**Published:** 2024-09-27

**Authors:** Chenxuan Bao, Qing Gao, Huayuan Xiang, Yuxuan Shen, Qiaoqiao Chen, Qianqian Gao, Yuanfei Cao, Mengyu Zhang, Wenyuan He, Lingxiang Mao

**Affiliations:** ^1^ Department of Laboratory Medicine, Affiliated Kunshan Hospital of Jiangsu University, Kunshan, Jiangsu, China; ^2^ Medical College, Yangzhou University, Yangzhou, Jiangsu, China

**Keywords:** human endogenous retroviruses (HERVs), virus infection, antiviral immunity, active immunity, HERV-K, HERV-W

## Abstract

The human genome harbors many endogenous retroviral elements, known as human endogenous retroviruses (HERVs), which have been integrated into the genome during evolution due to infections by exogenous retroviruses. Accounting for up to 8% of the human genome, HERVs are tightly regulated by the host and are implicated in various physiological and pathological processes. Aberrant expression of HERVs has been observed in numerous studies on exogenous viral infections. In this review, we focus on elucidating the potential roles of HERVs during various exogenous viral infections and further discuss their implications in antiviral immunity.

## 1 Introduction

Human endogenous retroviruses (HERVs) are remnants of exogenous retroviral infections that occurred millions of years ago in primates. Over time, most HERVs have lost their protein-coding capacity due to the degradation of open reading frames (ORFs) caused by the emergence of termination codons and frameshift mutations, resulting in the loss of their ability to replicate and infect (Magiorkinis et al., 2015). The naming and classification of HERVs are confusing, particularly HERV-K, which has been designated as HLM-2, HML-2, HTDV/HERV-K, HERV-K (HML-2), HERV-K, HERVK, or ERVK (Manghera et al., 2015). The current standard naming convention is based on the amino acid initials encoded by tRNAs primarily involved in their reverse transcription process, for example, HERV-K representing lysine-tRNA, HERV-H representing histidine-tRNA, and HERV-F representing phenylalanine-tRNA (Vargiu et al., 2016). Furthermore, based on the different ancestral exogenous retroviruses, HERVs are primarily classified into three classes: class I, gamma-like and epsilon-like; class II, beta-like; and class III, spuma-like (**Table 1**). Due to their origin, endogenous retroviruses exhibit similarities with exogenous retroviruses regarding genes, transcripts, and protein structures. Class I HERVs resemble gamma-retroviruses such as MLV and GalV. Class II HERVs are similar to typical beta-retroviruses like MMLV. Class III HERVs share affinity with lentiviruses or foamy viruses (Vargiu et al., 2016).

**Table 1:** Classification and clades of HERVs.

HERV genus	Probable genus	Type species	Supergroups	Canonical clade	Repbase identifiers
Class I (gamma-like, epsilon-like)	Gammaretrovirus and Epsilonretrovirus	Murine leukemia virus (MLV) Feline leukemia virus (FeLV) Walleye dermal sarcoma virus (WDSV)	MLLV	HERVT	HERVS71/LTR6
			HERVERI	HERVE	HERVE/LTR2
				HERV3	HERV3/LTR4
				HERV1	HERV1
				HERVI	HERVI/LTR10
			HERVW9	HERVW	HERV17/LTR17
				HERV9	HERV9/LTR12
			HERVIPADP	HERVIP	HERVIP10F/LTR10F
				HERVADP	HERVP71A_1/LTR71
			HERVHF	HERVH	HERVH/LTR7
				HERVH48	HERVH48I/MER48
				HERVFA	HERVFH19/LTR19
				HERVFB	HERVFH21/LTR21A
				HERVFC	HERV46I/LTR46
				LTR46	LTR46-in/LTR46
			HERVFRDlike	HERVFRD	ERV3-1-i/LTR58 MER50
				PRIMA41	PRIMA41/MER41
				HERV1_artiodact	NA
				PABL	PABL_BI/PABL_A, PABL_B
				HERV4	N/A
			HEPSI	HEPSI2	HUERSP1/LTR8
				HEPSI3	HUERSP2/LTR1_LTR28
				MER65	HUERSP3/LTR9
				PRIMA4	PRIMA4
			HUERSP	HUERSP1	HUERSP1/LTR8
				HUERSP2	HUERSP2/LTR1_LTR28
				HUERSP3	HUERSP3/LTR9
Class II (beta-like)	Betaretrovirus	Mouse mammary tumor virus (MMTV) Mason-Pfizer monkey virus (MPMV) Jaagsiekte sheep retrovirus (JSRV)	HML	HML1	HERVK14I/LTR14
				HML2	HERVK/LTR5
				HML3	HERVK9I/MER9
				HML4	HERVK13I/LTR13
				HML5	HERVK22/LTR22
				HML6	HERVK31/LTR3
				HML7	HERVK11DI/MER11D
				HML8	HERVK11I/MER11A
				HML9	HERVK(14C)/LTR14C
				HML10	HERVKC4/LTR14
Class III (spuma-like) including MaLR	Spumaretrovirus	Simian foamy virus (SFV)	HSERVIII	HERVL	HERVL (HERVL/MLT2)
				HERVS	HERVS (HERV18/LTR18)
Uncertain_Errantilike	Errantivirus	Gypsy retrovirus	Uncertain Errantilike	N/A	N/A

N/A, not applicable.

HERV-K belongs to the class II HERVs and represents a subtype characterized by the shortest integration time and the most intact sequence preservation (Jern et al., 2005). Its intact proviral sequence consists of two flanking long-terminal repeats (LTRs) and four ORFs, composing the *gag*, *pro*, *pol*, and *env* genes, which encode the four major viral proteins. In this case, *gag*, *pro*, and *pol* share the same transcript, and there are sequence duplications in all three ORFs, which are translated by two ribosomal frameshifts. The Gag protein has a full length of approximately 74 kDa. It undergoes subsequent processing by viral proteases to generate matrix protein (MA), capsid protein (CA), and nucleocapsid protein (NC), which participate in virus assembly and promote the maturation of viral particles (George et al., 2011). The pro gene encodes the viral protease responsible for cleaving the Gag protein (Schommer et al., 1996). At the junction between the *gag and pro* sequences, there is a region that encodes dUTPase, which reduces erroneous uracil insertion during DNA synthesis (Harris et al., 1997). The *pol* gene encodes the reverse transcriptase (RT), integrase (IN), and RNase H, which are essential for reverse transcription.

Env is translated via a separate transcript. The Env protein is typically synthesized as a precursor protein and, guided by the signal peptide (SP), undergoes cleavage by furin protease in the Golgi apparatus to generate two subunits: the surface unit (SU), which interacts with receptors on target cells, and the transmembrane unit (TM), which anchors to the cell membrane and facilitates membrane fusion (Henzy and Coffin, 2013; Gröger et al., 2020).

**Figure 1:**
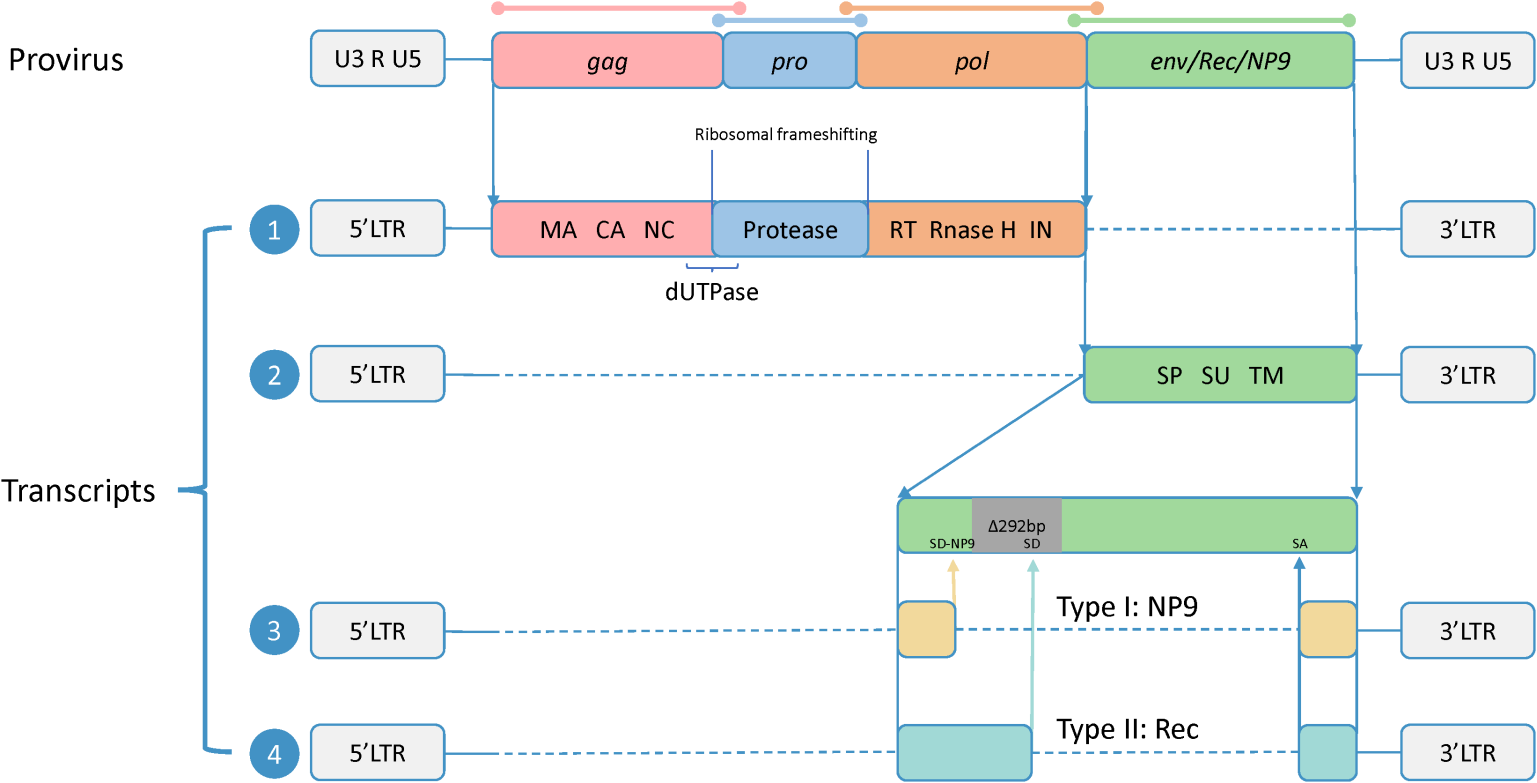
Proviral genomic structure and transcripts of HERV-K. The precursor sequence of HERV-K includes two flanking long-terminal repeats (LTRs) and four open reading frames (ORFs), and the sequences of four ORFs are overlapped (shown by colored lines). The two LTRs consist of U3 and U5 regions separated by an R fragment. The full-length HERV-K precursor sequence produces four transcripts. Transcript 1 contains three ORFs encoding Gag, Pro, and Pol, all sharing a common start codon. These ORFs overlap in their DNA sequences but not in their amino acid sequences, and they are translated via ribosomal frameshifting. The translation products are subsequently processed to generate the final functional subunits, including matrix (MA), capsid (CA), and nucleocapsid (NC) from Gag; dUTPase from the Gag-Pro junction; and reverse transcriptase (RT), RNase H, and integrase (IN) from Pol. Transcript 2 encodes Env, which is composed of the signal peptide (SP), surface (SU), and transmembrane (TM) subunits. Transcript 3 is produced from **t**ype 1, which has a 292-bp deletion at the pol**–**env junction. This deletion eliminates the original splice donor (SD) site, leading to the utilization of an upstream SD site (SD-NP9), and the transcript encodes NP9. Transcript 4 is produced from **t**ype 2, which has no nucleotide deletion and encodes the Rec.

Additionally, based on the presence or absence of a 292-bp deletion at the *pol–env* junction, HERV-K proviruses can be divided into two subtypes. Type-1 proviruses, characterized by the presence of the 292-bp deletion, lack a splice donor (SD) site necessary for Rec protein expression and instead utilize an alternative upstream SD site, resulting in the production of NP9 (Armbruester et al., 2002). Type-2 proviruses, lacking the 292-bp deletion, exhibit variable splicing patterns facilitated by SD and splice acceptor (SA) sites, leading to the transcription and translation of Rec proteins (Hohn et al., 2013). Rec and NP9, as accessory proteins, have functions that are not fully understood but may be involved in the transport of viral transcripts (Wodrich and Kräusslich, 2001; Blissenbach et al., 2010). However, both proteins have been confirmed to be associated with cancer, showing increased expression in various tumor tissues, and the related carcinogenic mechanisms have been studied to some extent (Armbruester et al., 2002; Denne et al., 2007; Chen et al., 2013; Hanke et al., 2013; Curty et al., 2020) (**Figure 1**).

The expression of HERV genes has been strictly regulated by the host organism over millions of years of coexistence, primarily through epigenetic mechanisms such as DNA methylation, deamination, heterochromatin silencing induced by histone modifications, and nucleosomal positioning (Maksakova et al., 2008; Hurst and Magiorkinis, 2017). Furthermore, the mRNA transcribed from HERVs can be significantly restricted by RNA instability modifications, RNA degradation mediated by host surveillance mechanisms, and species-specific piRNA-mediated RNA degradation (Ha et al., 2014; Chelmicki et al., 2021; Garland et al., 2022). Additionally, certain host genes and proteins can regulate HERV expression. For instance, APOBEC3F effectively inhibits the replication of HERV-K (Lee and Bieniasz, 2007), while APOBEC3G induces inactivating mutations in HERV-K, thereby suppressing its infection (Lee et al., 2008). MOV10 can efficiently control the activity of retrotransposons and regulate HERV expression (Arjan-Odedra et al., 2012). Members of the Krüppel-associated box zinc finger protein (KRAB-ZFP) family, such as ZFP809, initiate sequence-specific silencing of ERVs by recruiting heterochromatin-inducing complexes, with TRIM28 playing a crucial role in this process (Wolf et al., 2015; Taka et al., 2022). Moreover, the gene sequences of TRIM5 and TRIM22 overlap significantly with the LTRs of ERVs, potentially representing a form of genetic control (Diehl et al., 2013).

In somatic cells, although most HERVs are subject to epigenetic regulation, a certain number of HERV RNAs are ubiquitously transcribed. Specific expression patterns are observed in the brain, testes, blood, muscles, liver, and heart, with the central nervous system exhibiting the highest abundance, where approximately 3,000 specific HERV RNAs are transcribed (Burn et al., 2022; She et al., 2022). This expression is thought to result primarily from sequence overlap between HERVs and other genes, leading to their “expression” concurrently with the expression of these other genes. The physiological contributions of these normally expressed HERVs still need to be determined.

The epigenetic regulation of HERVs by the host may be impaired under pathological conditions, such as neoplastic and autoimmune diseases (Baudino et al., 2010; Rangel et al., 2022; Rivas et al., 2022). Exogenous factors like radiation, chemicals, and other viral infections can also activate HERV expression (Weiss, 2006). This review will primarily elucidate the interactions and roles of HERVs in exogenous viral infections.

## 2 The role of HERVs in exogenous viral infections

### 2.1 HERVs and HIV

The interaction between human immunodeficiency virus (HIV) and HERVs, both being retroviruses, has long been a focal point in HERV-related research. Firstly, several studies have shown that HIV-1 infection increases the transcription levels of HERVs (Contreras-Galindo et al., 2007; Bhardwaj et al., 2014; Li et al., 2021). However, no corresponding increase in viral particle production was observed (Bhardwaj et al., 2014). In contrast, a recent transcriptomic analysis of T cells post-HIV-1 infection shows a general downregulation of HERV transcription levels (Grandi et al., 2020). Some scholars attribute such contradictory results to contamination of genomic DNA and retrotransposon elements in the blood plasma and environment (Bhardwaj et al., 2014; Karamitros et al., 2016). Moreover, a microarray analysis of HIV-1-infected cell lines revealed significant differences in the activation of HERVs, particularly HERV-K, between new and persistent HIV-1 infections. It may indicate that new HIV-1 infection and persistent HIV-1 infection have different activation mechanisms for HERVs (Vincendeau et al., 2015).

The exact mechanism by which HIV infection activates HERVs remains unclear. Some suggest that the Tat protein of HIV-1 can activate the HERV-K LTR (van der Kuyl, 2012). High-throughput sequencing results indicate that HIV-1 Tat protein can significantly activate the transcription of 26 HERV-K subgroups while silencing 12 others (Gonzalez-Hernandez et al., 2014). Tat facilitates the transcription of HERV-K by inducing the loss of pericentromeric heterochromatin, thereby opening the chromatin structure (Contreras-Galindo et al., 2013). Additionally, the Vif protein of HIV-1 is believed to promote HERV expression by degrading the retrotransposon restriction factor APOBEC3G (Goila-Gaur and Strebel, 2008; Jones et al., 2012). Simultaneously, there is also a hypothesis suggesting that the activation of HERVs is an indirect result of secondary infections resulting from the collapse of the immune system following HIV infection (van der Kuyl, 2012).

In addition, HERVs and HIV share common complementary proteins, which may lead to the influence of HERVs on the replication process of HIV during infection. HERV-K Gag protein can co-assemble with HIV-1 Gag protein, but the resulting viral particles are immature and exhibit defects in release and infectivity (Monde et al., 2012, 2017). The HERV Env molecule can substitute or supplement the Env of HIV-1. Some researchers suggest that this pseudo-HIV-1 may have broader cellular tropism, which could explain why HIV is capable of infecting non-CD4^+^ target cells, leading to a more extensive and deeper infection (An et al., 2001, Brinzevich et al., 2014, Tang et al., 2020). However, some studies have found that Env molecules produced by HERV-K can inhibit the production of HIV-1 Gag protein, thereby reducing the generation of HIV-1 viral particles (Terry et al., 2017). Early views suggested that HERV-K Pro could substitute for drug-inhibited HIV-1 Pro in cleaving HIV-1 Gag protein. However, subsequent studies found that although HERV-K Pro can cleave HIV-1 Gag protein precursors, its cleavage is incorrect, leading to loss of HIV-1 infectivity (Towler et al., 1998; Padow et al., 2000). The *Pol* sequence encodes RT, IN, and RNase H. The IN of HERV-K10 has been shown to restore the biological activity of corresponding defective strains of HIV-1 but leads to the production of low-toxicity particles (Ogata et al., 1999). HERV-K Rec protein has a similar secondary structure to HIV-1 Rev protein, thus possessing similar regulatory effects. HIV-1 Rev can bind to HERV-K RcRE to activate HERV-K expression, not *vice versa*. HERV-K Rec does not activate HIV-1 expression and is not interchangeable (Sakai et al., 1990, Churchill et al., 2007, O'Carroll et al., 2020). As an exogenous lentivirus, HIV lacks the dUTPase commonly found in other lentiviruses, which results in a higher error rate during its replication process. The prevailing view suggests that HIV exhibits higher tolerance to uracil, but some scholars hypothesize that HIV may exploit HERV-K sequences to produce dUTPase (McIntosh and Haynes, 1996; Harris et al., 1997). Molecular biology studies of other primate viruses have found that HIV-related viruses contain the dUTPase gene, which may indirectly indicate the need for dUTPase by HIV (Gifford et al., 2008; Gilbert et al., 2009). In general, conflicting views regarding their functional implications have emerged (**Table 2**).

**Table 2:** Complementary **p**roteins of HIV and HERVs.

Component	HERVs	Effect	Mechanism	Ref.
Gag	HERV-K	Reducing release efficiency and infectivity of HIV	HERV-K co-assembles with HIV-1 via Gag to generate immature HIV-1 viral particles.	Monde et al. (2012, 2017)
Env	HERV-W	Substitute for HIV env	HERV-W Env glycoprotein can form pseudotypes with HIV-1 virions and may confer tropism for CD4-negative cells.	An et al. (2001)
	HERV-K18	Incorporation with HIV-1	HERV-K18 Env is specifically and efficiently incorporated into HIV-1 virions in a matrix-dependent fashion.	Brinzevich et al. (2014)
	HERV-W	Expanding the cellular tropism of HIV	Formation of HERV-W pseudotyped HIV allows infection of non-CD4-expressing cells.	Tang et al. (2020)
	HERV-K108	Interfering with HIV production	Residues within HERV-K108 Env inhibit HIV-1 production.	Terry et al. (2017)
Pro	HERV-K	Complement for HIV pro	HERV-K Pro complements HIV-1 Pro when the latter is inhibited.	Towler et al. (1998)
	HERV-K	Loss of infectivity of HIV	HERV-K protease misprocesses Gag and Pol proteins of HIV-1.	Padow et al. (2000)
Pol	HERV-K10	Substitute for HIV integrase	HERV-K10 integrase is able to recognize the dissimilar HIV-1 LTR.	Ogata et al. (1999)
Rec	HERV-K	Activate HERV expression	HIV-1 Rev can substitute for HERV-K Rec to bind to RcRE, thereby activating HERV-K.	O'Carroll et al. (2020)
dUTPase	HERV-K	Complementing the lack of HIV dUTPase	HIV may use the dUTPase enzyme encoded by the N-terminal part of the HERV-K protease gene.	McIntosh and Haynes (1996)

HERVs, human endogenous retroviruses; HIV, human immunodeficiency virus.

When HERVs are upregulated, the immune system mounts a corresponding response against them. Individuals infected with HIV-1 exhibit higher levels of antibodies against HERV-E, HERV-H, and HERV-K, and their T cells show stronger responses to HERV-H, HERV-K, HERV-W, and HERV-L. These antibody levels appear to reflect the efficacy of HIV-1 treatment in infected individuals, with lower antibody titers and T-cell response levels potentially indicating good control of HIV-1 infection. Thus, these antibodies have potential as biomarkers for detecting HIV-1 infection. For example, HERV-K Env becomes fully N-glycosylated following HIV-1 infection (van der Kuyl, 2012; Michaud et al., 2014a, 2014; de Mulder et al., 2017). Additionally, HERV LTRs are activated after HIV-1 infection, including the LTR12C sequence. This sequence has multiple antiviral functions, such as inhibiting the maturation of HIV-1 particles by blocking furin, an enzyme used to cleave viral proteins. It can also trigger antiviral responses by activating IRF1 or STAT1 (Chuong et al., 2016). Furthermore, the region around the LTR12C sequence is enriched with interferon-stimulated genes (ISGs), which may interact to enhance the body’s antiviral response (Srinivasachar Badarinarayan et al., 2020).

### 2.2 HERVs and SARS-CoV-2

Coronaviruses (CoVs) constitute a highly diverse family of enveloped, positive-sense single-stranded RNA viruses, which cause respiratory and intestinal infections in animals and humans. Seven coronaviruses are known to be infectious to humans. Human coronaviruses such as HCoV-229E, HCoV-OC43, HCoV-NL63, and HCoV-HKU1 are among the primary pathogens responsible for the “common cold,” typically causing only mild respiratory infections. In contrast, severe acute respiratory syndrome coronavirus, Middle East respiratory syndrome coronavirus, and severe acute respiratory syndrome coronavirus 2 (SARS-CoV-2) are highly pathogenic, capable of causing severe, life-threatening respiratory illnesses and lung damage (V'Kovski et al., 2021). SARS-CoV-2 is the pathogen responsible for the 2019 coronavirus disease (COVID-19), which escalated to a global pandemic in 2020. To date, it has resulted in 771 million infection cases and nearly 7 million deaths, causing severe loss of life and property worldwide and leading to significant socioeconomic disruption. Although most patients experience mild infections and can recover over time, a certain proportion of cases result in severe pneumonia, respiratory system failure, multiorgan failure, and even death. Approximately 10% of infected individuals suffer from the long-term chronic effects of long COVID following the acute infection phase (Wu and McGoogan, 2020; Davis et al., 2023; Zhang et al., 2024).

Studies observed the activation of HERV-W Env in peripheral blood leukocytes of patients infected with SARS-CoV-2, primarily involving CD4^+^ and CD8^+^ T cells. Considering the activation levels of HERV-W could predict respiratory outcomes during patient hospitalization, suggesting this could serve as an effective indicator for determining patient prognosis (Balestrieri et al., 2021; Garcia-Montojo and Nath, 2021). *In-vitro* studies demonstrated continuous stimulation of HERV-W Env expression in leukocytes by SARS-CoV-2, suggesting that the elevated expression of HERV-W Env in peripheral blood leukocytes of SARS-CoV-2-infected patients is a primary consequence of SARS-CoV-2 infection rather than a secondary result of inflammation (Balestrieri et al., 2021; Charvet et al., 2023). Kitsou et al. (2021) systematically analyzed the expression of various HERV family members in bronchoalveolar lavage fluid (BALF), among which HERV-FRD, HERV-H, HERV-K, and HERV-W were the most prominently upregulated subgroups. Concurrently, although HERV upregulation was observed in the BALF of patients with SARS-CoV-2 infection, it was not detected in peripheral blood mononuclear cells (PBMCs), indicating that the upregulation of HERVs in BALF may indeed be associated with local rather than systemic immune responses, further suggesting the critical role of HERVs in the inflammatory immune response induced by SARS-CoV-2 infection. Petrone et al. (2023) further discovered that SARS-CoV-2 infection leads to a widespread increase in the expression of HERVs and immune response mediators at nasal mucosa, which can be detected using conventional qRT-PCR. These indicators effectively predict the respiratory status and disease progression of infected patients. This suggests that the expression levels of HERVs combined with inflammatory factors can serve as effective and convenient early indicators for predicting the prognosis of infected patients. More studies further confirmed the elevated levels of HERVs in SARS-CoV-2-infected individuals (Giménez-Orenga et al., 2022; Guo et al., 2022; Simula et al., 2022; Temerozo et al., 2022; Grandi et al., 2023).

However, whether elevated HERVs can play a positive role in the organism’s resistance to SARS-CoV-2 infection is a matter of disagreement among scholars. Simula et al. (2022) reported an increased presence of IFN autoantibodies in correlation with HERV-W-env autoantibodies in COVID-19 patients, suggesting an association between the two, indicating that the overexpression of HERV-W Env impairs the immune response. On the other hand, Guo et al. (2022) found that HERV-K-related genes were highly expressed in both *in-vivo* and *in-vitro* experiments of SARS-CoV-2 infection, and these were positively correlated with IFN-related gene expression in moderate and severe cases, suggesting that the high-level expression of HERV-K stimulates an increase in IFN in patients, thereby enhancing the immune response. Undoubtedly, HERVs have a significant activating effect on IFN, which may be an important antiviral immune response of the body. However, the abnormal activation of both can potentially lead to the production of related autoantibodies, which may cause interference and damage to the patient’s immune system.

Grandi et al. first reported that HERV levels are dynamically modulated during SARS-CoV-2 infection by high-throughput sequencing of the HERV locus in the PBMCs of COVID-19 patients (Grandi et al., 2023). They suggested that HERVs are more likely to act as an immune sentinel rather than the main force against infection in the immune response to SARS-CoV-2 infection. Elevated HERV levels in adult infection cases typically indicate a poor prognosis (Balestrieri et al., 2021; Kitsou et al., 2021), but in an analysis of children’s resistance to SARS-CoV-2 infection, the results differ. HERV-related protein levels were higher in mild pediatric cases, while severe cases had no upregulation. Furthermore, the results showed a close correlation between HERV levels and the expression levels of antiviral factors such as IFN-I, IFN-II, SYN1, SYN2, TRIM28, and SETDB1, indicating that HERVs may enhance innate immune responses when children’s immune function is still intact and can mount a normal immune response to viral infection (Tovo et al., 2021). The opposite results in pediatric cases may suggest that HERVs play different roles in innate and acquired immune responses.

In an analysis of SARS-CoV-2-regulated transposable elements (TEs), it was revealed that SARS-CoV-2 infection induces widespread TE activation, leading to the upregulation of HERV family members. These differentiated TEs are also associated with transcription factors and pioneer transcription factors involved in immune responses, which may explain the underlying logic of HERV upregulation and modulation of immune responses after SARS-CoV-2 infection (Marston et al., 2021). As a newly emerged virus, further research is needed on the interaction between SARS-CoV-2 and HERVs. Current data generally agree that dysregulated levels of HERVs during SARS-CoV-2 infection correlate with patient prognosis. In adult cases, elevated HERVs usually indicate a worsening of the disease prognosis and thus have the potential to be used as prognostic and therapeutic targets (Desingu and Nagarajan, 2023).

### 2.3 HERVs and influenza viruses

Influenza viruses belong to the family Orthomyxoviridae and are single-stranded, negative-sense, segmented RNA viruses. The main types causing seasonal influenza are influenza A and B. Influenza A viruses (IAVs) can be further classified into numerous subtypes based on the structure of their surface hemagglutinin (HA) and neuraminidase (NA) proteins. Despite decades of monitoring and interventions, seasonal influenza cases continue to occur worldwide, leading to significant consumption of healthcare resources and causing a substantial number of deaths (Petrova and Russell, 2018). Influenza virus infections are typically accompanied by inflammatory solid responses, which are crucial for controlling virus replication but can also lead to lung damage, morbidity, and mortality (Tavares et al., 2017).

As early as 2006, Nellåker et al. (2006) observed that IAV infection promotes the transactivation of a subset containing HERV-W elements. In 2014, the same research group again validated this phenomenon and further discussed the underlying mechanisms. They found that IAVs can enhance the transcription of HERV-W elements, including the syncytin-1 sequence, by upregulating the transcription factor glial cells missing 1 (Li et al., 2014). In a study on IAV-triggered antiviral responses, researchers found that IAV infection leads to a decrease in SUMO-modified TRIM28, which is closely associated with the self-suppression of HERVs, resulting in high-level transcription of HERV elements. The high-level transcription of HERV sequences can form dsRNA, enhancing the typical IFN-mediated immune response involving RIG-I, MAVS, TBK1, and JAK1 (Schmidt et al., 2019). The authors also found that IAV has strategies to counteract this immune response; they observed that the IAV NS1 protein effectively antagonizes the formation of dsRNA, thus significantly blocking this pathway and weakening the occurrence of IFN-related immune responses (Schmidt et al., 2019). With the application of omics research techniques, the role of HERVs in antiviral responses has been further elucidated. Transcriptomic analysis has shown that influenza virus infection can induce the transcription of many abnormal HERV loci, thereby stimulating the upregulation of neighboring genes. Interestingly, these neighboring genes are almost all ISGs (Wang et al., 2021; Liu et al., 2022). The analysis results suggest that HERVs play a significant role in the organism’s antiviral immune response.

### 2.4 HERVs and herpesviruses

Herpesviruses, enveloped viruses with a double-stranded DNA genome, represent one of the most prevalent viral families among humans, infecting approximately 80%–90% of the adult population worldwide. Over a hundred herpesviruses have been identified, of which eight are known to infect humans: herpes simplex virus 1 (HSV-1), herpes simplex virus 2 (HSV-2), varicella-zoster virus (VZV), Epstein–Barr virus (EBV), human cytomegalovirus (HCMV), human herpesvirus 6 (HHV-6), human herpesvirus 7 (HHV-7), and Kaposi’s sarcoma-associated herpesvirus (KSHV) (Christensen, 2007). The frequent detection of viral DNA (vDNA) from herpes viruses like EBV, VSV, and HHV-6 in multiple sclerosis (MS) patients has spurred decades of research into the potential impacts of herpesviruses on the course of MS (Khalesi et al., 2023). Among these investigations, the interplay between HERVs and herpesviruses has emerged as a focal point.

Anomalous activation of HERV-K18 Env has been detected in patients infected with various herpesviruses, serving as a superantigen molecule (sAg) that triggers aberrant immune responses closely linked to MS pathogenesis. These viruses include EBV, HHV-6A, HHV-6B, and HSV-1 (Sutkowski et al., 2001; Kwun et al., 2002; Hsiao et al., 2006; Tai et al., 2009; Turcanova et al., 2009; Ilse et al., 2022). Herpesviruses activate HERV-K18 through multiple pathways. For instance, EBV interacts with the CD21 molecule on the surface of B cells, leading to HERV-K18 expression (Hsiao et al., 2006). Similarly, HHV-6B mediates the interaction between its glycoprotein H and the CD46 molecule on APC cells. Both of these interactions occur independently of viral replication (Turcanova et al., 2009). Moreover, the replication process of these viruses can also activate HERV-K18 sAg expression. For instance, Sutkowski et al. (2004; Hsiao et al., 2009) found that the EBV Latent Membrane Protein 2A (LMP-2A) phosphorylates its immunoreceptor tyrosine-based activation motif, thus transactivating HERV-K18 expression. Kwun et al. (2002) discovered that the immediate early protein 0 of HSV-1 upregulates the activity of the cellular transcription factor AP-1, thereby enhancing the transactivation of HERV-K18 LTR. Additionally, herpesvirus infections, akin to influenza virus infections, induce IFN-mediated immune responses that can also trigger HERV-K18 sAg activation (Turcanova et al., 2009), potentially constituting a component of the host immune response. However, recent murine studies validating the sAg nature of HERV-K18 have failed to observe the onset of MS in animal models (Ilse et al., 2022).

Apart from HERV-K, members of the HERV-W family are also implicated in MS pathogenesis, with MS-associated retrovirus (MSRV) being the first identified HERV-W member (Perron et al., 1997). The activation of HERV-W by herpesvirus infections has been well-documented. As early as 2003, Lee et al. (2003) observed that the immediate early protein 1 of HSV-1 enhances the activity of Octamer Binding Transcription Factor 1, which binds to the HERV-W LTR region, thereby stimulating targeted gene expression. More evidence has indicated that HSV-1, EBV, HHV-6A/B, and CMV all have activating effects on HERV-W (Lee et al., 2003; Ruprecht et al., 2006; Mameli et al., 2012; Petersen et al., 2012; Mameli et al., 2013; Bergallo et al., 2018; Charvet et al., 2018; Meier et al., 2021; Pérez-Pérez et al., 2021, 2022; Cossu et al., 2023). Several research groups have observed concurrent elevation of HERV-K and HERV-W in their experiments, suggesting similar triggering mechanisms and potential common pathways (Alvarez-Lafuente et al., 2008; Bergallo et al., 2018). For example, Brudek et al. (2007) found that inactivated HSV-1, HHV-6, and VZV viruses can widely activate the levels of reverse transcriptase in lymphocytes of MS patients, indicating a global increase in the expression levels of all endogenous retroviruses within cells. Similarly, Leung et al. (2018) found that EBV can globally activate enhancers and promoters within HERV LTRs in B lymphocytes, thereby disrupting host gene regulatory networks at a genome-wide level.

In a recent study, Mameli et al. (2013) proposed that herpesviruses such as EBV induce the onset of MS through cascading inflammation. Persistent and frequent activation of EBV and other herpesviruses within the central nervous system triggers local inflammation, leading to myelin damage. With the combined effects of sEV, Epstein–Barr nuclear antigen 1(EBNA1)–DNA complexes, EBNA1–IgG–complement immune complexes, HERV-W/K sAg elements, and the early reactivation induced by other herpesvirus infections, the inflammatory cascade continuously amplifies, ultimately resulting in MS onset. HERVs play a crucial role in this process, and restricting them could effectively reduce inflammation levels (Meier et al., 2021). This may also explain why, in animal experiments, a mere increase in HERVs can trigger a certain degree of activation of the host autoimmunity but is insufficient to cause significant pathological changes at the macroscopic level (Ilse et al., 2022).

In addition to MS, KSHV and EBV are also important tumor viruses. KSHV, also known as HHV-8, is associated with multiple human malignancies, primarily causing Kaposi’s sarcoma (KS) and primary effusion lymphoma (PEL) in immunocompromised patients (Chang et al., 1994; Cesarman et al., 1995; Alomari and Totonchy, 2020). Due to the close association between KSHV and HIV infections, there may be some latent connections between KSHV and HERVs. Dai et al. (2018) observed a significant increase in HERV-K transcription levels in HIV-positive patients’ PBMCs and PEL tumor cells, possibly due to the potential viral proteins Latency-Associated Nuclear Antigen and the viral FLIP inhibitory protein of KSHV activating HERV-K through mitogen-activated protein kinase, nuclear factor κB (NF-κB), and other signaling pathways, as well as cell transcription factors like specificity protein 1. HERV-K proteins, particularly Rec and NP9, may promote the development of KSHV-related malignancies. Furthermore, the authors observed a positive correlation between HERV-K Env protein expression levels and vascular endothelial growth factor, which is considered a driving factor in the progression of endothelial cells infected by KSHV into an invasive phenotype (Qian et al., 2007). Hence, HERVs may play a critical role in tumorigenesis during KSHV infection.

EBV is also associated with various cancers. As mentioned earlier, EBV may transactivate the expression of HERV-K18 through LMP-1/2A and binding to CD21 on B cells (Hsiao et al., 2006, 2009). The activated NP9 appears to regulate EBV gene expression in infected cells. Gross et al. (2011) detected a strong upregulation of NP9 protein in EBV^+^ cell lines, which strongly downregulated the activity of EBNA2 and suppressed vDNA generation. This may represent a host defense mechanism or an adaptive mechanism of EBV to protect receptor cells, achieving immune evasion and long-term latency.

### 2.5 HERVs and hepatitis viruses

Human hepatitis viruses are classified into types A, B, C, D, E, and G. Although HBV is a dsDNA virus, it primarily replicates through reverse transcription of an RNA intermediate. Its polymerase shares amino acid homology with the retroviral reverse transcriptase and interacts with the core protein of the hepatitis C virus (HCV) (Chen et al., 2001). Sequence analysis reveals some similarities between the HBV genome and HERVs (Miller and Robinson, 1986). Liu et al. (2017) found that hepatitis B virus X protein (HBx) can induce the overexpression of HERV-W Env in hepatocellular carcinoma cells, which can be inhibited by NF-κB, suggesting HBV may overexpress HERV-W Env in hepatocellular carcinoma cells HBx via NF-κB. Elevated levels of HERV-K transcription were also detected in chronic HCV-infected patients, which correlated with liver function decline and treatment outcomes (Weber et al., 2021). The conventional HCV treatment drugs are ineffective against the elevation of HERVs. This results in high levels of HERV-related components, including Rec and NP9, in patients’ bodies following HCV infection. This may explain why patients remain at high risk of developing HCV-related cancers even after chronic HCV infection is suppressed (Tovo et al., 2020).

## 3 Role of HERVs in antiviral defense

### 3.1 The role of HERVs in antiviral immunity

In the interactions above between HERVs and various viral infections, it is clear that HERVs can mediate antiviral immune responses in the body. However, few studies have delved into the specific mechanisms involved. Currently, several immune pathways have been researched to some extent. The NF-κB signaling pathway has been shown to be mediated by HERVs. HERV-K dUTPase proteins can mediate NF-κB activation by activating Toll-like receptor 2 (Ariza and Williams, 2011). Additionally, in mouse experiments, ERV-derived lncRNA can competitively bind to restriction factors to promote the activity of the NF-κB family protein RelA (Zhou et al., 2019). HERVs may also be involved in activating the cGAS–STING signaling pathway, where the transcription of ERVs in mouse keratinocytes promotes the induction of commensal-specific T cells through the cGAS–STING pathway (Lima-Junior et al., 2021).

### 3.2 Antiviral effects of HERV-derived proteins

After viral infection of a cell, it can prevent infection by other related viruses. Therefore, some scholars speculate that vertebrates may utilize endogenous retroviruses within their genomes to protect against exogenous retroviral infections (Malfavon-Borja and Feschotte, 2015). Such effects have been previously demonstrated in mice and cats (Ikeda and Odaka, 1984; Best et al., 1997; Malfavon-Borja and Feschotte, 2015). Similarly, the Env protein of HERVs may reduce the infection levels of exogenous viruses by competitively binding to the target cell receptors of these viruses. In recent studies, HERV-H48 Env Suppressyn and HERV-W Env Syncytin-1 can competitively bind to ASCT2, a cell surface amino acid transporter, thereby blocking the infection of viruses like type D retroviruses that depend on this transport pathway (Frank et al., 2022; Khare et al., 2024). Additionally, the auxiliary protein Rec from HERV-K has been shown to increase the levels of IFITM1 on the cell surface, thereby triggering a precise antiviral response (Grow et al., 2015). This may explain why none of the three subgroups of HERVs (epsilon-like, beta-like, and spuma-like) have infectious exogenous retroviral counterparts, whereas exogenous retroviruses that infect humans (lentiviruses and deltaretroviruses) do not have corresponding HERV subgroups (van der Kuyl, 2012).

### 3.3 Application of HERVs in active immunity and treatment against HIV

Given the structural and functional similarity to HIV, HERVs have long been considered as potential targets for making effective vaccines against HIV. Garrison et al. (2007) observed that HIV-1 infection can stimulate HERV-positive CD8^+^ T cells, which can effectively control plasma viral load and lyse cells presenting target peptides. SenGupta et al. (2011) found that anti-HERV immunity can play a role during chronic HIV-1 infection. Anti-HERV CD8^+^ T cells can respond to cells infected with HIV-1 in a Vif-dependent manner, and *in-vitro* experiments have shown that these cells can eliminate cells infected with HIV-1, HIV-2, and SIV (Jones et al., 2012). Studies have demonstrated that antibodies against the γ retrovirus TM neutralize HIV-1 gp41 (Denner, 2013). Subsequent research showed that antibodies against the HERV-K Env TM subunit could also kill cells infected with HIV-1 through antibody-dependent cellular cytotoxicity mechanisms in *in-vitro* experiments (Michaud et al., 2014b). These antiviral effects may also be relevant in vertical transmission patients (Tandon et al., 2011). However, animal experiments have not been as straightforward. In an experiment involving rhesus macaques, inoculation with endogenous retroviral elements (EREs) did not protect the experimental animals from SIV infection. Specifically, ERE-specific T cells failed to undergo memory expansion *in vivo* after infection with SIVsmE660 (Sheppard et al., 2014). This experiment also revealed the species specificity of HIV and HERVs, complicating the establishment of relevant animal models.

In recent years, some scholars have proposed an alternative therapeutic hypothesis: to mimic and accelerate the body’s natural regulatory process of HERVs over a million-year timeline to achieve sustained silencing or even permanent inactivation of HIV in the patient’s body. Factors such as KRAB and TRIM, which play significant roles in the silencing of HERVs, may potentially achieve specific silencing of HIV-1 proviruses through similar mechanisms. This necessitates further research into the regulatory mechanisms of HERVs and the targeted silencing mechanisms of HIV (Lyons et al., 2023). Additionally, the “shock and kill” approach has been proposed as a strategy for an HIV cure. This is an opposite therapeutic strategy, where dormant viruses are activated through medication to induce the body’s immune system to eliminate these latent proviruses. However, these drugs may cause off-target effects and other pathological phenomena. Due to the homology between HERVs and HIV, it has been speculated that HERVs may interfere with this treatment process. In studies on histone deacetylase inhibitors, this drug does not induce the expression of HERV-K, HERV-W, or HERV-FRD, but they do significantly induce the expression of LTR12 elements of the HERV-9 family, which may be of concern to researchers developing these therapies (Hurst et al., 2016; White et al., 2018).

Due to the stable presence of HERV sequences in the human genome, their good compatibility with the host, and the potential cross-immunity between HERVs and HIV, active immunity against HERVs may serve as an important avenue for HIV vaccine design or post-HIV infection treatment.

## 4 Conclusions and perspectives

In summary, although initially considered genomic junk, increasing research has revealed that these foreign genes, which occupy nearly 8% of the human genome, play a significant role in the pathophysiology of human diseases. While the involvement of HERVs in various cancers and autoimmune diseases has been extensively and intensively studied, as a virus in its own right, its interaction with exogenous viral infections has not received sufficient attention. It is increasingly apparent that HERVs are intricately linked to viral replication, host antiviral immunity, and the sequelae of many kinds of exogenous viral infections. However, the precise role they play during exogenous viral infections still needs to be discovered. Whether they act as foes or allies, their consistent upregulation following various viral infections is well-established. This characteristic may enable them to serve as auxiliary markers for diagnosing and prognosis of infectious diseases. Furthermore, as members of the human genome, HERVs harbor the potential to serve as effective targets or vectors for antiviral therapies and antiviral active immunizations. In conclusion, a deeper understanding of endogenous retroviruses present in our genome is warranted to elucidate their potential interactions with exogenous viruses.
